# Quantification of Allyl Methyl Sulfide, Allyl Methyl Sulfoxide, and Allyl Methyl Sulfone in Human Milk and Urine After Ingestion of Cooked and Roasted Garlic

**DOI:** 10.3389/fnut.2020.565496

**Published:** 2020-09-18

**Authors:** Wen Qin, Katrin Huber, Moritz Popp, Patrick Bauer, Andrea Buettner, Constanze Sharapa, Laura Scheffler, Helene M. Loos

**Affiliations:** ^1^Department of Chemistry and Pharmacy, Chair of Aroma and Smell Research, Friedrich-Alexander-Universität Erlangen-Nürnberg, Erlangen, Germany; ^2^Fraunhofer Institute for Process Engineering and Packaging IVV, Freising, Germany

**Keywords:** gas chromatography-mass spectrometry, allyl methyl sulfide, allyl methyl sulfoxide, allyl methyl sulfone, human urine, human milk, garlic, odor

## Abstract

Due to its characteristic flavor and positive effects on human health, garlic is a highly valued food ingredient. Consumption of garlic alters the quality of body odors, which may in some instances hinder social interaction but be beneficial in other contexts, as it is assumed to contribute to early flavor learning in the breastfeeding context, for example. In previous work, allyl methyl sulfide (AMS) has been identified as the major odor-active metabolite in urine and milk, being excreted together with the odorless metabolites allyl methyl sulfoxide (AMSO) and allyl methyl sulfone (AMSO_2_) after ingestion of raw garlic. The present work aimed to elucidate whether commonly used culinary thermal processing steps influence the excretion profiles of garlic-derived compounds. To this aim, urine (*n* = 6) and milk (*n* = 4) samples were donated before and after ingestion of roasted and cooked garlic and investigated by gas chromatography-olfactometry/mass spectrometry, and, in the case of milk, by aroma profile analysis. The concentrations of AMS, AMSO, and AMSO_2_ were determined by stable isotope dilution assays. Sensory evaluations revealed that a garlic-like odor was perceivable in milk samples donated after ingestion of roasted and cooked garlic. Besides AMS, AMSO, and AMSO_2_, no other odor-active or odorless compounds related to the ingestion of roasted or cooked garlic were detected in the urine and milk samples. Maximum concentrations of the metabolites were detected around 1–2 h after garlic intake. In some cases, a second maximum occurred around 6 h after ingestion of garlic. The cooking procedure led to a more important reduction of metabolite concentrations than the roasting procedure. These findings suggest that intake of processed garlic leads to a transfer of odor-active and odorless metabolites into milk, which contributes to early flavor learning during breastfeeding and may also have a physiological effect on the infant.

## Introduction

Garlic (*Allium sativum*) is a common food ingredient, which is thought to originate from Central Asia where it has been cultivated for thousands of years. Today, garlic is distributed all over the world and is popular for its characteristic flavor. Next to its culinary use, garlic is known for its beneficial health effects and was already used as remedy in ancient Egypt, Greece, Italy, China and India (1). More recent research revealed sulfur-containing compounds as an important class of bioactive substances of garlic, having antimicrobial, anti-inflammatory, antioxidant, immunomodulatory, antihypertensive, antihyperlipidemic, cancer-preventive, and other physiological effects (2–5). Due to these promising health benefits, a range of garlic supplements were developed by industry, generally on the basis of garlic powder or extract. The physiological effects of these different supplements were often investigated but seldom compared to the effects of garlic as it would be consumed in common dietary intake conditions (6).

It is a well-known phenomenon that ingestion of garlic and garlic products alters body odors like the odors of breath (7–9), urine (10), breast milk (11, 12), and amniotic fluid (13). Breath odor is influenced by garlic-derived odor-active compounds such as allyl mercaptan, diallyl disulfide and allyl methyl sulfide (AMS), the latter being emitted continuously from the circulatory system for several hours (7–9, 14). Garlic-derived breath odor is generally considered unpleasant and can interfere with social interaction, which is why several attempts have been made to reduce this odor (15). Breast milk (hereafter: milk) also emits a garlic-like odor after garlic consumption by the mother (11, 12, 16). Milk odor influences breastfeeding interactions (17). Moreover, milk is usually the sole food of newborns in the first months and its flavor can affect the nursling's feeding behavior (18, 19) and also later food preferences (20). To examine how garlic intake influences milk odor and composition can thus provide valuable information for breastfeeding women. The garlic-like odor of milk has been reported to be most intensive about 2 h after ingestion, and to make infants spend significantly more time breast-feeding, yet without influencing milk intake (11, 21). Recently, we identified AMS to be responsible for the garlic-like odor of milk after consumption of raw garlic (12, 16). Accordingly, this compound, which is physiologically active in adults (22), may be ingested by the infant when breastfed after the mother ingested raw garlic. We further demonstrated that two odorless metabolites, namely allyl methyl sulfoxide (AMSO) and allyl methyl sulfone (AMSO_2_) are excreted into milk along with AMS (12, 16). These metabolites are also excreted into urine (10, 23), together with other substances which are however considered being of minor quantitative importance in realistic consumption scenarios (10, 24).

The majority of the population ingests garlic as a food ingredient. Common culinary processing steps of garlic like roasting, frying or cooking may influence the contents and the bioavailability of its bioactive constituents and also the intensity and quality of garlic-derived body odors (6, 25, 26). Quantitative data on the excretion of AMS, AMSO, and AMSO_2_ into milk and urine are available in relation to the intake of raw garlic. However, the impact of different culinary treatments on the resulting body odors and the excretion pattern of these metabolites has not been investigated to date. Therefore, this present work aimed to elucidate the volatile garlic-derived metabolites in milk and urine samples obtained after consumption of roasted and cooked garlic. Moreover, the metabolites AMS, AMSO, and AMSO_2_ were to be quantified in these samples over a time interval of up to 8 h in the case of milk and up to 24 h in the case of urine.

## Materials and Methods

### Ethical Approval of the Study

This study was carried out in accordance with the recommendations of the Declaration of Helsinki. The protocol (registration no. 49_13B), which included a detailed description of the sampling procedure as well as the sensory-analytical investigation of the samples, was approved by the Ethical Committee of Friedrich-Alexander-Universität Erlangen-Nürnberg. All subjects gave written informed consent before participating in the study. Withdrawing from the study was possible at any time without any negative consequences.

### Chemicals

Dichloromethane (DCM) and anhydrous sodium sulfate (Na_2_SO_4_) were supplied by VWR (Darmstadt, Germany), AMS and AMSO_2_ were obtained from Aldrich (Steinheim, Germany). The isotopically labeled standards ^2^H_3_-AMS, ^2^H_3_-AMSO and ^2^H_3_-AMSO_2_, as well as AMSO were supplied by aromaLAB GmbH (Freising, Germany). DCM was freshly distilled prior to use.

### Preparation of Cooked and Roasted Garlic

Fresh garlic was purchased from a local supermarket in Erlangen, Germany, peeled, and cut into 3 mm cubes using a garlic cutter (Genius GmbH, Limburg/Lahn, Germany). To obtain roasted garlic, 3 g of fresh, cut garlic were roasted under continuous stirring for 3 min in a pan pre-heated at 180°C, as determined via a Voltcraft^®^ infrared thermometer (Conrad Electronic SE, Hirschau, Germany). To obtain cooked garlic, 3 g of fresh garlic were put into 250 mL of boiling water (Evian, Danone S.A., Paris, France), and cooked for 10 min with a closed lid. The water was ingested together with the cooked garlic.

### Participants

Participants were healthy and did not report any known illness or metabolic disorder. All mothers had normal breast milk production. Two days before the sampling day, all participants started to avoid foods containing sulfurous compounds, namely garlic, onion, ramson, chives, cabbage, leek, and olives. They further provided dietary records of these 3 days (according to the form provided in the Supplementary Material).

#### Participants in the Qualitative Sensory-Analytical Screening

Four (three men, one woman) and three (two males, one woman) participants (age range: 23–26 years) donated urine sets after ingestion of roasted and cooked garlic, respectively. Three mothers donated milk sets after ingestion of roasted garlic, and three mothers donated milk sets after ingestion of cooked garlic (age range: 31–38 years).

#### Participants in the Quantitative Experiments

Six participants (five men, one woman; age range: 23–26 years) donated urine sets after ingestion of roasted garlic and six participants (four men, two women; age range: 23–27 years) donated urine sets after ingestion of cooked garlic. Five of the overall seven participants took part in both the cooked and the roasted garlic study. Four mothers donated milk sets for both the study with roasted and the study with cooked garlic. The age of the mothers ranged between 31 and 33 years.

### Urine and Milk Samples

#### Donation of Urine Samples

Urine samples were collected in brown glass bottles before, and half an hour, 1, 2, 4, 6, 8, and 24 h after garlic ingestion. If the participant donated more than 50 mL at one point in time, a maximum of 50 mL was retained for the sample workup (the total volume was not determined). Urine volumes used for the sample workup varied between 9 and 50 mL. The urine samples were either immediately used or stored up to 24 h at 4°C or up to 1 month at −80°C before use.

#### Donation of Milk Samples

Milk samples were obtained before and at three points in time after garlic ingestion. The times of the donations after garlic ingestion were determined by the individual breastfeeding rhythm of each mother-infant dyad, and hence varied between mothers. In general, the samples were donated 2, 4, and 6 h after garlic ingestion. The milk was collected by the mothers before or after feeding their infant, using an electric or manual milk pump, and transferred into brown glass bottles. The donated milk volume ranged between 13 and 43 mL. The milk samples were transported from the mothers' homes to the laboratory to be sensorily evaluated (see section Aroma Profile Analysis of Milk Samples), and were then either immediately used or stored up to 24 h at 4°C or up to 2 weeks at −80°C before use. The milk samples were not cooled during the transport (5–30 min) to allow for sensory evaluation of milk samples at room temperature.

### Sensory, Analytical, and Sensory-Analytical Methods

#### Aroma Profile Analysis of Milk Samples

The aroma profiles of the milk samples were determined by three to six trained panelists (age range: 23–33 years). The training consisted in weekly sensory evaluations of varying reference solutions, allowing the panelists to learn the flavor language of the Chair of Aroma and Smell Research, and to correctly identify and describe individual odorants. To evaluate the aroma of the milk samples, the panelists were asked to rate the perceived intensities of odor attributes on a scale ranging from 0 (no perception) to 3 (strong perception), also allowing 0.5-increments. The milk samples were presented to the panelists in brown glass bottles (100 mL, 40 mm aperture) immediately after arriving from the mother's home. The attributes were selected according to previous investigations (12, 16): hay-like, fishy, fatty, rancid, sweaty, metallic, grassy-green, sweet, egg white-like, lactic, butter-like, and garlic-like.

#### Isolation of Odorants and Metabolites From Urine and Milk Samples

Freshly distilled DCM was added to the samples in a ratio of 1:2 (DCM/body fluid, v/v) and stirred for 30 min at room temperature. Solvent assisted flavor evaporation [SAFE; (27)] at 50°C and 10^−4^ mbar was subsequently applied to isolate the volatiles from other matrix components. Before finishing the SAFE, the glassware and the SAFE apparatus were rinsed twice with 10 mL DCM to achieve complete transfer of the respective volatiles. After thawing, the organic phase was separated from the aqueous phase, dried over anhydrous Na_2_SO_4_, filtered, and concentrated to a total volume of 100 μL by means of Vigreux and micro-distillation at 50°C. The distillates were then used for further analyses.

#### High-Resolution Gas Chromatography-Olfactometry

High-resolution gas chromatography-olfactometry (HRGC-O) was performed by two trained panelists (one woman, age: 23 years; one man, age: 25 years) with a Trace Ultra GC (Thermo Fisher Scientific, Waltham, MA, USA), equipped with either a DB-FFAP or a DB-5 capillary column (30 m × 0.32 mm, film thickness 0.25 μm; J & W Scientific, Agilent Technologies, Santa Clara, CA, USA). The cold-on-column manual injection technique was used to transfer an aliquot of 2 μL of the distillates to an uncoated precolumn (3 m × 0.32 mm). This technique prevents thermal degradation of labile compounds, such as sulfur compounds. The flow rate of the carrier gas helium was 2.0 mL/min. The temperature was held at 40°C for 7 min, and raised to 240°C (FFAP) or 250°C (DB-5) at 8°C/min and was held for 5 min. The effluent was split at the end of the capillary by a Y-type quick-seal glass splitter into a sniffing port (270°C) and a flame ionization detector (FID; 250°C) using deactivated, uncoated fused silica capillaries (i.d. 0.32 mm). Linear retention indices (RIs) were calculated according to van den Dool and Kratz (28). Comparative HRGC-O analyses were performed to compare the overall odor composition of samples obtained before and after garlic intake.

#### High-Resolution Gas Chromatography-Mass Spectrometry (HRGC-MS)

An Agilent MSD quadrupole system (GC 7890A and MSD 5975C; Agilent Technologies) equipped with a Multipurpose Sampler MPS 2, a CIS 4 injection system (both Gerstel GmbH & Co. KG, Mühlheim an der Ruhr, Germany), and a DB-FFAP capillary column (30 m × 0.25 mm, film thickness 0.25 μm; J & W Scientific, Agilent Technologies) was used for GC-MS analyses. The carrier gas flow (helium) was 1 mL/min. Mass spectra were recorded at 70 eV in full scan mode (m/z range 30–350) and/or SIM mode (see section Quantification of AMS, AMSO, and AMSO_2_). The oven temperature was first held at 40°C for 7 min, then raised at 8°C/min to 240°C and held for 8 min (16, 23). Injection volumes were 2 μL in the automated cold-on-column technique.

#### Two-Dimensional High-Resolution Gas Chromatography-Mass Spectrometry (Heart-Cut)

The two-dimensional HRGC-MS system consisted of two Agilent 7890 B GCs in combination with an Agilent 5977 B MS (Agilent Technologies). The GCs were connected by a CTS 1 cryotrap system, and equipped with a Multipurpose Sampler MPS 2 and a CIS 4 injection system; furthermore, the first oven was equipped with a multi-column switching system MCS (all Gerstel GmbH & Co. KG, Mühlheim an der Ruhr, Germany). A DB-5 capillary column (30 m × 0.32 mm, film thickness 0.25 μm; J & W Scientific, Agilent Technologies). was used in the first oven, and a DB-FFAP capillary column was installed in the second oven (30 m × 0.25 mm, film thickness 0.25 μm; J & W Scientific, Agilent Technologies). The flow rate of the carrier gas helium was 2.5 mL/min in the first GC and 1 mL/min in the second GC. Temperature was held at 40°C for 7 (first oven) and 8 (second oven) minutes, then raised at 20°C/min to 300°C (first oven) and 240°C (second oven) and held for 5 min (16, 23). The automated cold-on-column injection was applied with 2 μL injection volume. The effluent in the first oven was split into an FID (250°C), a sniffing port (270°C), and, during heart-cut, into the cryotrap at a 70:30 ratio. The effluent in the second oven was split into a sniffing port (270°C) and the MS, by using deactivated, uncoated fused silica capillaries. Mass spectra were recorded at 70 eV in full scan mode (m/z range 30–100) and/or in SIM mode (see section Quantification of AMS, AMSO, and AMSO_2_).

#### Quantification of AMS, AMSO, and AMSO_2_

Quantification of AMS, AMSO, and AMSO_2_ was carried out via stable isotope dilution assay [SIDA; (29)], as previously described by Scheffler et al. (16, 23). By using this approach, potential losses of the volatile compounds during the distillation process are taken into account. The amounts of the target metabolites were semi-quantitatively evaluated in the GC-O and GC-MS screenings (cf. 3.1.1; data not shown) to determine the amounts of isotopically labeled standard to be added to the samples. The standards (^2^H_3_-AMS, ^2^H_3_-AMSO and ^2^H_3_-AMSO_2_) were dissolved in DCM and added to the body fluids which were then stirred for 10 min. Subsequently, the samples were prepared for analysis according to section Isolation of Odorants and Metabolites From Urine and Milk Samples. The quantification of AMS was carried out in SIM mode (m/z 88 and m/z 91), using the two-dimensional HRGC-MS system. The quantification of AMSO and AMSO_2_ was achieved with the HRGC-MS system in SIM mode (m/z 104 and m/z 107 for AMSO and ^2^H_3_-AMSO; m/z 120 and m/z 123 for AMSO_2_ and ^2^H_3_-AMSO_2_). LOD and LOQ of this method have previously been determined for both urine and milk (16, 23). The concentrations were calculated using the calibrations obtained by measuring defined mixtures of the analyte and the isotopically labeled standard. Based on five-point-calibration lines, equations used for calculation of concentrations were, for AMS: *y* = 0.9231x-0.0016 (*R*^2^ = 0.9988), for AMSO: *y* = 0.7364x + 0.0409 (*R*^2^ = 0.9993), for AMSO_2_: *y* = 0.5412x + 0.0934 (*R*^2^ = 0.9986).

#### Determination of Creatinine in Urine

The creatinine content in the urine samples was determined via the Jaffé reaction, using a creatinine kit (Labor+Technik Eberhard Lehmann GmbH, Berlin, Germany). Under basic conditions, creatinine and picric acid yield a yellow complex which was photometrically quantified at 492 nm.

## Results

### Qualitative Sensory-Analytical Screening

#### Gas Chromatographic-Olfactometric Screening of Urine and Milk Samples

##### Urine samples

Comparative GC-O analyses revealed the presence of one garlic-like smelling compound at RI 715 (DB-5) in distillates obtained from urine samples donated after consumption of roasted garlic, which was identified as AMS. No other odors related to garlic consumption were perceived at the sniffing port. In distillates obtained from urine samples donated in the cooked garlic study, no odor-active compounds related to garlic consumption were detected by GC-O.

##### Milk samples

A garlic-like smelling odorant was detected at RI 715 (DB-5) in distillates obtained from milk samples donated after ingestion of roasted garlic, which was identified as AMS. No other odor-active compounds related to garlic intake were present in the distillates. In distillates obtained from milk samples donated in the cooked garlic study, no odor-active compounds related to garlic consumption were detected by GC-O.

#### Gas Chromatographic-Mass Spectrometric Screening of Urine and Milk Samples

##### Urine samples

A screening using HRGC-MS did not reveal any quantitatively abundant metabolites related to roasted or cooked garlic ingestion in distillates obtained from urine samples. Nonetheless, a targeted analysis via HRGC-GC-MS showed that the metabolites AMS, AMSO, and AMSO_2_ were present in distillates obtained from urine samples after ingestion of roasted and cooked garlic.

##### Milk samples

No quantitatively abundant metabolites related to ingestion of roasted or cooked garlic were evident in HRGC-MS analyses. A targeted analysis using HRGC-GC-MS revealed the presence of AMS, AMSO, and AMSO_2_ in distillates obtained from milk samples after intake of roasted garlic. For cooked garlic, AMS and AMSO_2_ were only detected in one of the three sample sets, whereas AMSO was not detected.

### Quantitative Analysis of AMS, AMSO, and AMSO_2_ in Urine and Milk

#### Urine Samples

Detailed results of all investigated samples, including the volume of urine samples, concentrations of metabolites, and concentrations of creatinine, are provided in Supplementary Table 1.

##### Roasted garlic

**Figure 1:**
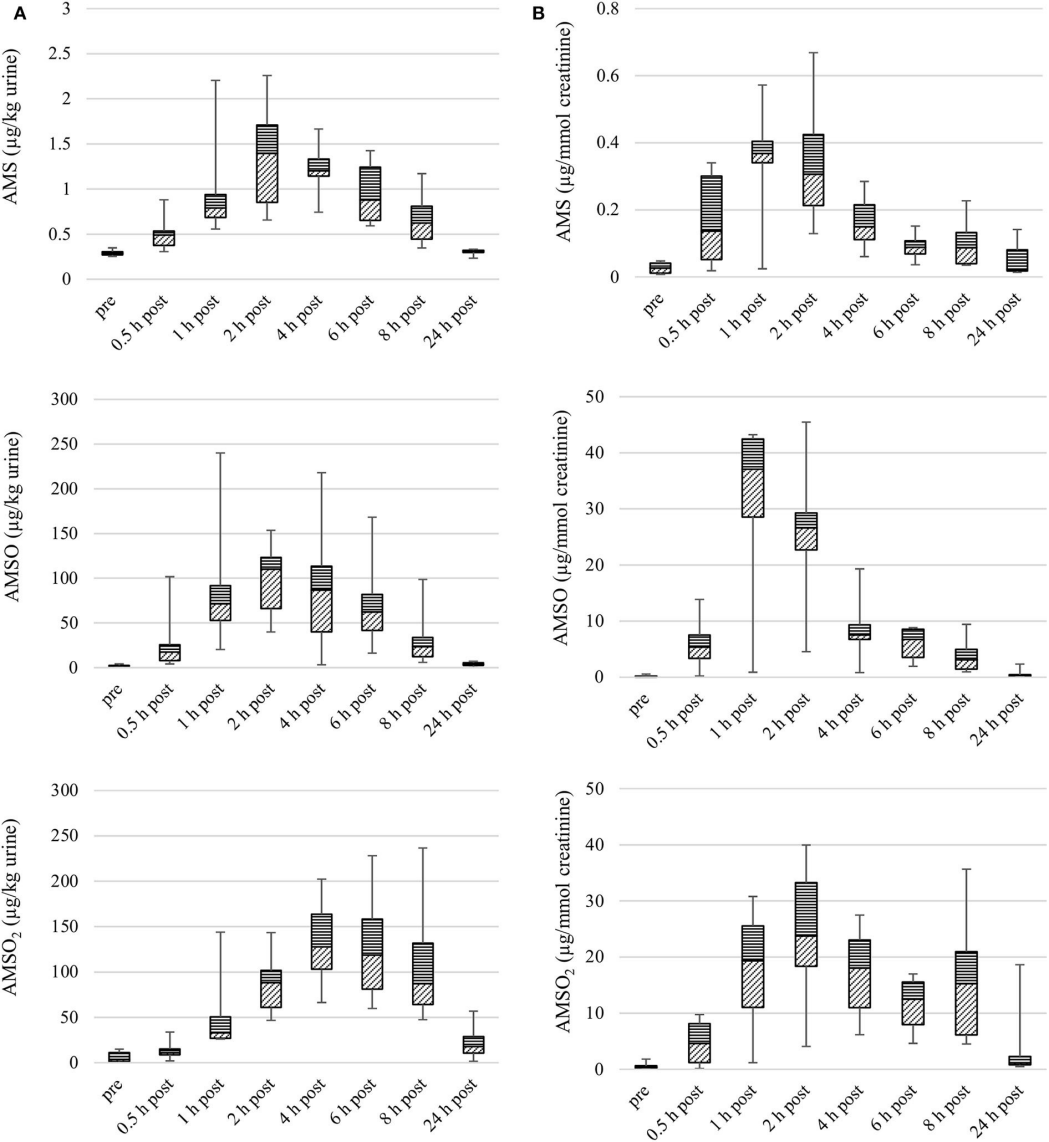
Box plot (median value, markers at minimum and maximum metabolite concentration, box percentile 25–75) of garlic metabolite (AMS, AMSO, and AMSO_2_) concentrations in urine, in μg/kg **(A)** and μg/mmol creatinine **(B)**. Data from six participants, urine samples obtained before (“pre”), or after (“0.5 h post” to “24 h post”) intake of roasted garlic.

The concentrations of AMS, AMSO, and AMSO_2_ in urine samples obtained before and after ingestion of roasted garlic are shown in Figure 1. The temporal excretion pattern appears slightly different when concentrations are calculated in μg/kg urine compared to values expressed as μg/mmol creatinine. This is especially striking for AMSO and AMSO_2_. Maximum concentrations of these metabolites occur later when calculated in μg/kg urine compared to values in μg/mmol creatinine. Furthermore, a second maximum of metabolite concentrations becomes evident for values in μg/mmol creatinine, but not for those in μg/kg urine. In the following, we will refer to concentrations based on the creatinine content of the urine samples to account for potential dilution effects caused by varying water intake and resorption of the participants.

The median concentration of AMS peaked 1 h after intake of roasted garlic (median: 0.4 μg/mmol creatinine). The following sample still contained an elevated level of AMS (median: 0.3 μg/mmol creatinine). Samples collected 4, 6, 8, and 24 h after garlic intake contained lower concentrations of AMS. The individual maximum concentrations measured in urine samples obtained from different participants varied between 0.1 and 0.7 μg/mmol creatinine. The maximal median concentration of AMSO, 37.1 μg/mmol creatinine, was reached 1 h after garlic intake. The sample collected at 2 h after garlic intake still contained an elevated level of AMSO (median: 26.6 μg/mmol creatinine), whereas median concentrations were in the range of 0.2 to 7.5 μg/mmol creatinine in the following urine samples. The individual maximum concentrations of AMSO, as measured in urine sample series obtained from the six participants, varied between 6.6 and 45.5 μg/mmol creatinine.

The median concentration of AMSO_2_ peaked 2 h after garlic ingestion (median: 23.8 μg/mmol creatinine). Elevated levels of AMSO_2_ were also evident in samples obtained 1, 4, 6, and 8 h after garlic intake, with a second apex occurring 8 h after garlic intake (median: 15.3 μg/mmol creatinine). The individual maximum concentrations in the six sample series ranged between 9.2 and 40.0 μg/mmol creatinine.

##### Cooked garlic

**Figure 2:**
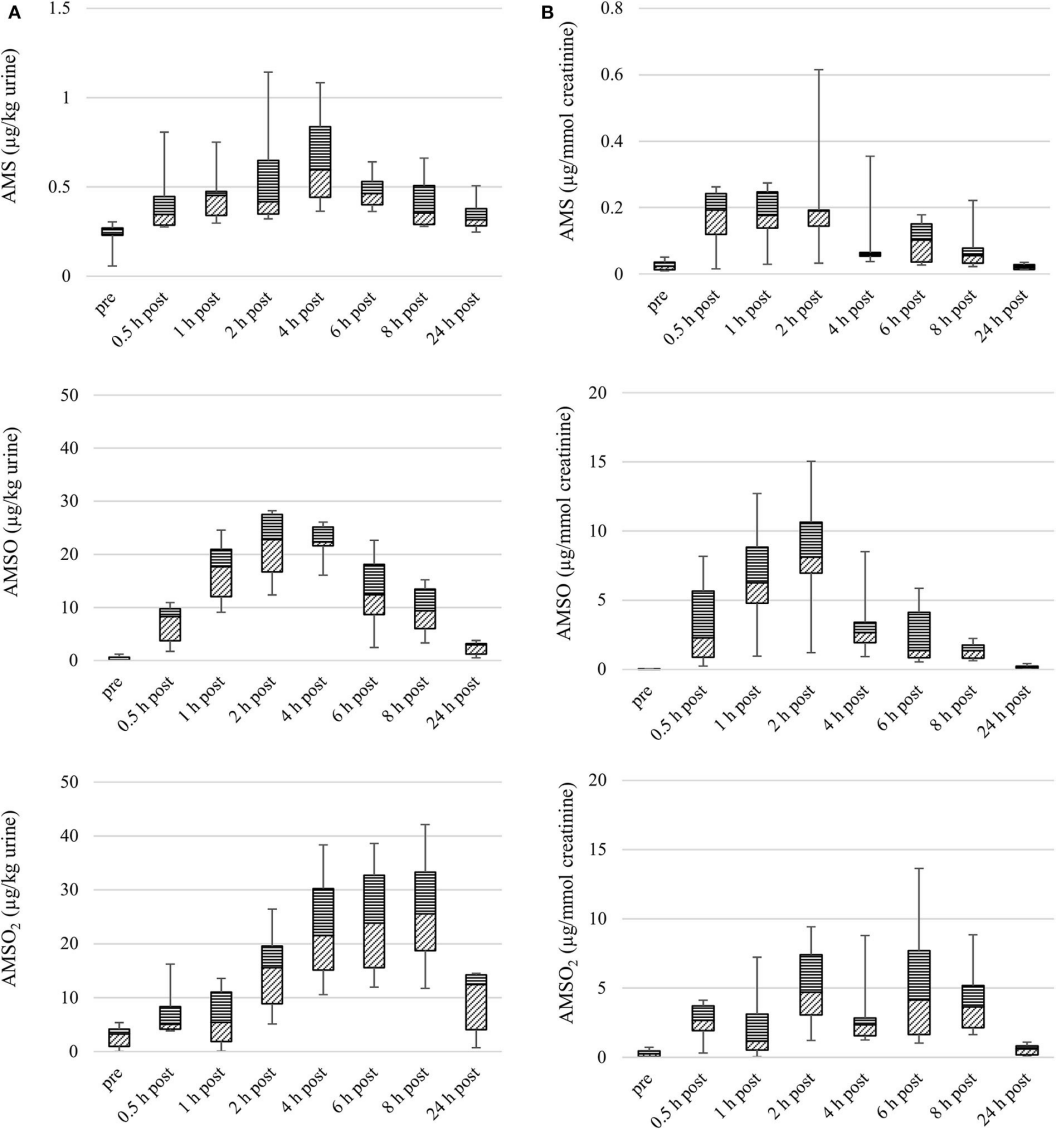
Box plot (median value, markers at minimum and maximum metabolite concentration, box percentile 25–75) of garlic metabolite (AMS, AMSO, and AMSO_2_) concentrations in urine, in μg/kg **(A)** and μg/mmol creatinine **(B)**. Data from six participants, urine samples obtained before (“pre”), or after (“0.5 h post” to “24 h post”) intake of cooked garlic.

The concentrations of AMS, AMSO, and AMSO_2_ in urine samples obtained before and after ingestion of cooked garlic are shown as boxplots in Figure 2. As already observed for results obtained with roasted garlic, the temporal excretion profiles were different for concentrations expressed as μg/kg urine compared to those expressed as μg/mmol creatinine. In the following, we will refer to concentrations taking the creatinine content of urine into account.

The median concentration of AMS peaked 0.5 h after intake of cooked garlic (median: 0.2 μg/mmol creatinine). Similar median concentrations were detected in samples collected 1 and 2 h after garlic intake. A second apex occurred 6 h after garlic intake (median: 0.1 μg/mmol creatinine). The individual maximum concentrations in the six sample series ranged from 0.04 to 0.6 μg/mmol creatinine.

The maximal median concentration of AMSO was reached 2 h after garlic intake (median: 8.1 μg/mmol creatinine). An elevated level of AMSO was further detected in samples obtained 1 h after garlic intake (median: 6.3 μg/mmol creatinine). In samples obtained 4, 6, 8, and 24 h after garlic intake, lower concentrations were determined. Among the six sample series, individual maximum concentrations of AMSO ranged between 1.2 and 15.0 μg/mmol creatinine. The median concentration of AMSO_2_ peaked 2 h after garlic intake (median: 4.7 μg/mmol creatinine), with a second apex occurring 6 h after garlic intake (median: 4.1 μg/mmol creatinine). The maximum concentrations determined in the individual sample series varied between 1.7 and 13.6 μg/mmol creatinine.

##### Individual temporal profiles of excretion

**Figure 3:**
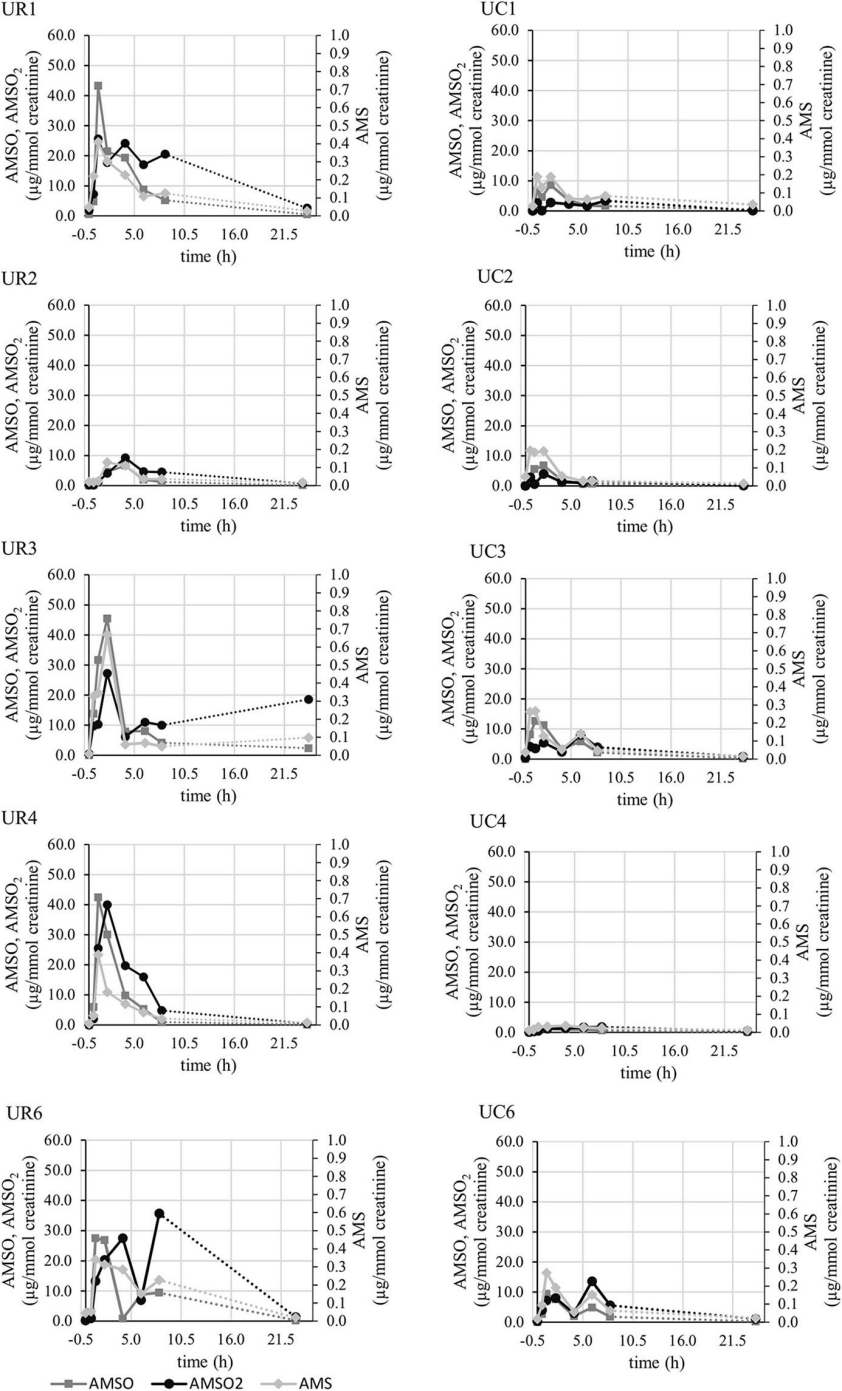
Concentrations (in μg/mmol creatinine) of garlic metabolites AMS (light gray line), AMSO (gray line), and AMSO_2_ (black line) detected in urine collected before and after ingestion of roasted garlic (left side, “UR”) or cooked garlic (right side, “UC”) in the 5 participants taking part in both studies.

The individual temporal profiles of garlic metabolite excretion via urine are shown in Figure 3 for those five persons who participated twice (once for roasted, once for cooked garlic). Whereas, two apex concentrations became clearly apparent for two participants (UR3, UC3, UR6, UC6), this was less evident for the other sample series. The duration until maximum metabolite concentrations slightly differed between participants. For instance, the maximum concentration of AMS was reached 1 h (UR1, UR4, UR6), or 2 h (UR2, UR3, UR5) after garlic intake. By comparison of excretion profiles regarding roasted and cooked garlic, it becomes further evident that the metabolite concentrations in urine obtained after ingestion of cooked garlic were generally by a factor of 2, or more, lower than those obtained after roasted garlic intake. However, it is noteworthy that exceptionally low metabolite concentrations have been detected in sample series UR2 (roasted garlic), which are comparable to those obtained in sample series UC2 (cooked garlic).

#### Milk Samples

**Figure 4:**
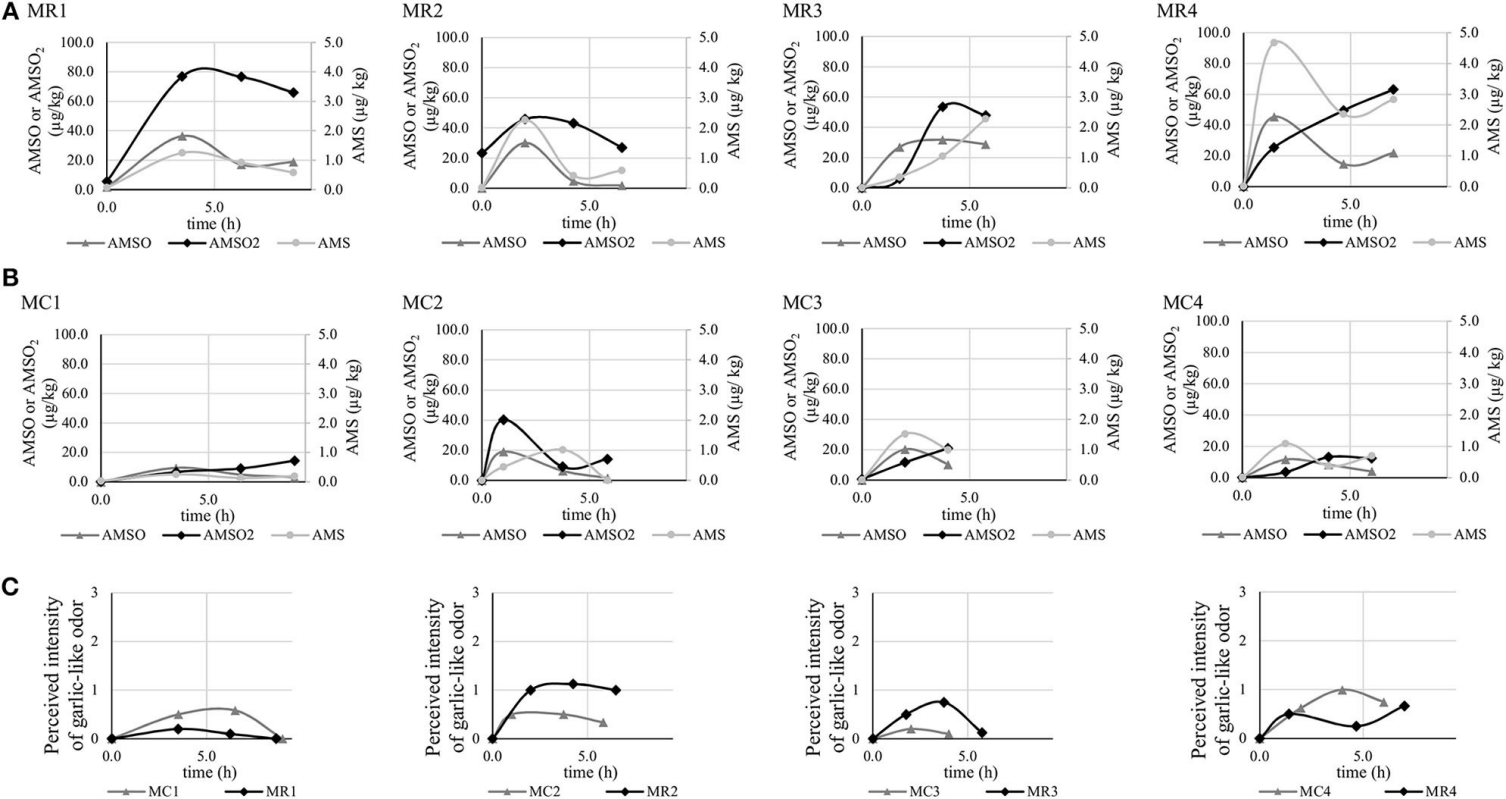
Concentrations (in μg/kg) of garlic metabolites AMS, AMSO, and AMSO_2_ detected in milk collected before and after ingestion of roasted **(A)** and cooked **(B)** garlic in 4 participants. Perceived intensity of a garlic-like odor in the milk samples **(C)**.

Detailed results of all investigated samples, including the mass of milk samples and concentrations of metabolites, are provided in Supplementary Table 1. The temporal profiles of garlic metabolite excretion via milk are shown in Figure 4.

The individual maximum concentrations of AMS ranged from 1.2 (MR1) to 4.7 μg/kg (MR4) and from 0.3 (MC1) to 1.5 μg/kg (MC3) after ingestion of roasted and cooked garlic, respectively. The peak concentrations were generally detected in the first or second sample donated after garlic intake, i.e., around 1–3 h after garlic intake. However, there is one exception (MR3). In this sample series, the concentration of AMS increased from the first to the fourth sample. It is further worth mentioning that in some cases, the concentration of AMS raised again in the fourth sample, i.e., around 5–7 h after garlic intake.

The maximum concentrations of AMSO varied between 30.0 (MR2) and 45.3 μg/kg (MR4) after ingestion of roasted garlic, and between 9.5 (MC1) and 20.3 μg/kg (MC3) after ingestion of cooked garlic. They were generally reached around 1–4 h after garlic intake. In two sample series (MR1, MR4), concentrations of AMSO increased again in the fourth sample.

The maximum concentrations of AMSO_2_ ranged from 45.8 (MR2) to 76.7 μg/kg (MR1) and from 13.1 (MC4) to 40.3 μg/kg (MC2) after ingestion of roasted and cooked garlic, respectively. These were generally reached within the first 4 h after garlic intake, with the exception of sample series MR4, MC1, and MC3, which evinced a continuous increase of AMSO_2_ after garlic intake, at least within the respective time frame of milk donation. It is noteworthy that AMSO_2_ was present in the baseline sample of series MR2.

### Aroma Profile Analysis of Milk Samples

The ratings of general odor attributes associated with milk (see section Aroma Profile Analysis of Milk Samples) corresponded to previous reports [e.g., (10)] and are therefore not reported in detail. A garlic-like odor was not perceived in any of the samples obtained before garlic ingestion (see Figure 4, bottom row). However, all samples collected after ingestion of roasted or cooked garlic emitted a garlic-like smell, which was on average rated to be of low to medium intensity.

## Discussion

### Temporal Profiles of Metabolite Excretion

In previous studies, Scheffler and colleagues identified AMS, AMSO, and AMSO_2_ as garlic-derived metabolites (10, 12) and revealed their temporal excretion profiles in urine and milk after consumption of 3 g of raw garlic (16, 23) and 10 g of raw ramson (30). In the present work, we showed that AMS, AMSO, and AMSO_2_ are likewise excreted into urine and milk after consumption of roasted and cooked garlic. The metabolite concentrations (μg/mmol creatinine) peaked around 0.5–2 h (urine) and 1–4 h (milk) after garlic consumption. In some sample series, a second maximum appeared about 6 h after garlic ingestion. Similar temporal excretion profiles were observed after ingestion of raw garlic and ramson (16, 23, 30), indicating that roasting or cooking garlic prior to ingestion does not change the temporal excretion profiles of metabolites.

As already discussed by Scheffler et al. (10, 12, 16, 23, 30), AMS, AMSO and AMSO_2_ derive from allyl thiosulfinates. Allicin is the predominant allyl thiosulfinate in garlic (2) and is rapidly metabolized to allyl mercaptan as an intermediate metabolite (31, 32). AMS is then formed by methylation of allyl mercaptan. Degradation products of allicin, for instance polysulfides, are also metabolized to AMS (22). Eventually, AMS is converted into two derivatives, AMSO and AMSO_2_, which are more persistent (33). The appearance of a second maximum in metabolite concentrations might be explained by different adsorption sites within the gastrointestinal system, as proposed by Scheffler et al. (23). Digestive processes start in the mouth. Foods are crushed into small pieces and enzymes in the saliva break down a small number of compounds (34, 35), and it is well-known that some molecules can be absorbed in the human mouth and esophagus (36, 37). Allicin and related compounds can easily pass cell membranes (38) and are thought to be absorbed rapidly and almost quantitatively (22). However, Suarez et al. (9) report that AMS was not detected in urine when garlic was chewed but not ingested. Therefore, it seems unlikely that absorption in the mouth contributes to the here observed double peak phenomenon. An alternative explanation is that the garlic-related molecules are digested and absorbed primarily in the stomach, but that the second main absorption location is the small intestine. A further possible explanation is that the two peaks represent two different sources of, or metabolic pathways to, allyl mercaptan. Whereas, allicin is rapidly metabolized, other compounds such as diallyl disulfide or S-allyl cysteine are degraded more slowly (6, 32). It is interesting to note that the double peak phenomenon appears to be specific to individual participants in the present study [but see Scheffler et al. (30) for different temporal courses in one individual]. It might thus be related to individual expression profiles of enzymes involved in digestive and metabolic processes. From the food protocols of the respective participants, no hints were obtained on potential diet-related influencing factors. However, it cannot be excluded that part of the food protocols may have been filled out incorrectly or not detailed enough to trace back intake of “hidden” garlic or onion products. Thus, with the data at hand, the underlying reason remains unclear and future experiments appear necessary to resolve this issue.

To quantify the target metabolites in urine samples independent of dilution effects due to dietary water intake, creatinine was used as reference molecule. Creatinine is a byproduct of muscle metabolism and formed at a constant rate (39). It is excreted via urine and its concentration can be used to normalize concentrations of other metabolites excreted in urine (40–44). From Figures 1, 2 it becomes evident that the temporal profiles of excretion differ according to whether creatinine concentration was considered or not. Notably for AMSO_2_, the maximum concentration occurred apparently later when its concentrations were calculated in units of μg/kg urine. A similar trend became evident in earlier work (23). This trend might result from temporal patterns regarding the participants' intake of water, which might be influenced by day time or by the time within the experiment. For instance, some participants might drink more water in the beginning to ensure donation of urine samples, and refrain from doing so later in the experiment. However, it is not entirely clear why this effect would be more impactful for the apparent temporal excretion profiles of AMSO_2_ than for the two other metabolites.

### Impact of Thermal Processing on Metabolite Concentrations

As outlined above, the temporal profiles of metabolite excretion were similar for roasted and cooked garlic and were in line with earlier results obtained on raw garlic. However, the metabolite concentrations differed. Information on individual maximum metabolite concentrations in milk and urine samples obtained after ingestion of raw (16, 23), roasted and cooked garlic is compiled in Table 1. On average, these concentrations decrease from raw to roasted, and from roasted to cooked garlic, around two- (milk) to 3- to 4-fold (urine) in each case. There appears to be an exception regarding AMS in milk, for which a higher average maximum concentration was determined with roasted garlic than with raw garlic. However, this outcome might be skewed because of the low number of participating mothers in the present study and would need to be corroborated in further studies. In sum, the concentrations of the three target metabolites were generally more elevated in samples obtained after consumption of raw garlic compared to samples obtained after consumption of roasted or cooked garlic. This indicates that the concentration of molecules yielding AMS, AMSO and AMSO_2_ is significantly reduced after thermal treatment.

**Table 1:** Average (range) of maximum metabolite concentrations detected in urine and milk samples after ingestion of raw, roasted, and cooked garlic.

	**Maximum concentrations in urine sample series [μg/mmol creatinine]**		
	**Raw garlic^a^, *n* = 19**	**Roasted garlic, *n* = 6**	**Cooked garlic, *n* = 6**
AMS	0.8 (0.3–2.3)	0.4 (0.1–0.7)	0.3 (0–0.6)
AMSO	136.3 (27.6–344.1)	34.6 (6.6–45.5)	8.9 (1.2–15.0)
AMSO_2_	102.2 (32.1–284.7)	28.8 (9.2–40.0)	6.7 (1.7–13.6)
	**Maximum concentrations in milk sample series [μg/kg]**		
	**Raw garlic^a^, *n* = 18**	**Roasted garlic**, ***n*** **=** **4**	**Cooked garlic**, ***n*** **=** **4**
AMS	2.0 (0.6–4.1)	2.6 (1.2–4.7)	1.0 (0.3–1.5)
AMSO	78.2 (30.0–144.9)	35.9 (30.0–45.3)	15.2 (9.5–20.3)
AMSO_2_	108.5 (48.7–200.3)	59.8 (45.8–76.7)	22.2 (13.1–40.3)

a *According to Scheffler et al. (16, 23)*.

There are manifold ways of preparing garlic for culinary use. For instance, it may be chopped or sliced, or used uncrushed. It may be cooked or roasted or fried, and the temperature and duration of these thermal treatments may vary just as other ingredients being added during these processes. Thiosulfinates are unstable and easily transformed to polysulfides, especially under higher temperature (2). Moreover, there is evidence that other flavor precursors (e.g., deoxyalliin, γ-glutamyl-*S*-allylcysteine) are also transformed to polysulfides under heating treatments (45, 46). The extent to which thiosulfinates are built and decomposed depends largely on the respective processing conditions (2, 6, 26). In the present study, the garlic was first sliced into 3 mm-cubes using a garlic cutter. It is expected that this process generates much less allicin than chopping: according to Lawson and Hunsaker (6) about 3% of alliin is biotransformed by alliinase upon cutting garlic into 3 mm cubes. In a second step, 3 g of the sliced garlic were roasted for 3 min at 180°C (roasted garlic) or cooked for 10 min in 250 mL of boiling water (cooked garlic). Thermal inactivation of alliinase significantly reduces the amount of allicin available for absorption. At the same time, degradation products of allicin, e.g., sulfides, are more rapidly formed during heat treatment. These might, however, also be lost during prolonged heating since many of these organosulfur compounds are highly volatile. Inhibition of allicin formation and loss of polysulfides are considered to be the main reasons why the concentrations of AMS, AMSO and AMSO_2_ in urine and breast milk were lower after consuming roasted and cooked garlic, compared with raw garlic. Locatelli et al. (26) investigated the impact of different pre-cooking and cooking treatments on the amounts of allicin, ajoene, 2-vinyl-4H-1,3-dithiin, diallyl sulfide, diallyl disulfide, and diallyl trisulfide in garlic. Amongst others, the authors confirmed that the presence of oil is an important influencing factor favoring decomposition of allicin and formation of (poly)sulfides, dithiins and ajoenes: allicin was not detected any more after stir-frying sliced garlic (2 mm cubes) for 2 min. In contrast, allicin was still detected when sliced garlic was cooked in water (simmering, for 15 min: 0.3 μmol/g; rolling boil, for 6 min: 1.0 μmol/g; as compared to raw sliced garlic: 15.0 μmol/g), and we therefore assume that the cooking procedure applied in the present study did not lead to a complete degradation of allicin. Cavagnaro et al. (25) assessed the effect of heating crushed and uncrushed garlic in an oven at 200°C or boiling it in water, respectively, for different durations (1, 3, 6, 10, and 20 min). The results highlight that the duration of thermal treatment is an important influencing factor on alliinase activity and allicin concentrations. The different durations of the here applied roasting and cooking procedures (3 vs. 10 min) are thus one possible reason why lower metabolite concentrations were detected after ingesting cooked garlic. Other possible explanations are the temperature difference between the two conditions (180 vs. 100°C) or the absence vs. presence of water during the heating process. Recently, Lawson and Hunsaker (6) reported that allicin bioequivalence (as determined via AMS concentrations in exhaled breath) was by a factor two higher in roasted compared to cooked garlic, and trace this phenomenon back to higher allyl polysulfide concentrations in roasted garlic. In their study, the duration of cooking (4 or 45 min) did not influence AMS concentrations, nor was this the case for the roasting temperature (oven temperature 215 and 160°C for 60 and 30 min, respectively). However, Lawson and Hunsaker did not slice the garlic before the thermal treatment, and the garlic water was not consumed by the participants, which prevents direct comparison of the two studies. Lawson and Hunsaker (6) also state that allicin bioequivalence was surprisingly high in alliinase-inhibited garlic preparations such as roasted, cooked or acidified garlic and that the conversion of γ-glutamyl-*S*-allylcysteine, *S*-allylcysteine and alliin to AMS was enhanced in the absence of alliinase, the underlying mechanism still being unclear. For future studies on the quantitative excretion patterns of AMS, AMSO, and AMSO_2_, it is thus recommendable to quantify allicin, allyl polysulfides, and other potential metabolite precursors in the ingested garlic foods to elucidate the impact of different culinary processing steps on these target compounds, and correlate it to the excretion of metabolites.

Among the 12 urine sample series, UR2 and UC4 contained distinctively lower metabolite concentrations compared to the other sample series. According to the food protocols, both volunteers had a normal diet. For UC4, the creatinine values were, however, on average higher than in the other urine sample series. For UR2, this is less striking, but the creatinine values appear also slightly elevated for this urine series. We are not aware of any problems which would have occurred during the determination of creatinine concentrations. Future studies on excretion patterns of AMS, AMSO, and AMSO_2_ might reveal whether individual variation in metabolism accounts for this phenomenon or whether water intake influences distribution and excretion processes of garlic-derived metabolites in the human body. To fully explore potential inter-individual differences in metabolism and excretion of garlic-derived compounds, a higher number of participants and gender-balanced study population would be needed which could not be afforded in the present study. To this aim, future work may be designed to use rapid and automatized detection techniques of the target metabolites.

### AMS: an Odor-Active Metabolite in Milk

After garlic consumption, a low-intensity yet distinct garlic-like odor was perceived in each of the eight milk sample series. The relatively strongest odor was perceived between 1 and 4 h after garlic consumption (except MR4 at 7 h and MC1 at 6.5 h), which is in line with previous work on raw garlic (12, 16). Even though the metabolites were excreted in lower concentrations after ingestion of roasted and cooked garlic, the intensity of the garlic-like odor in the milk samples was not lower compared to earlier work on raw garlic. Likewise, the odor intensities were comparable when mothers ate roasted or cooked garlic, in spite of higher concentrations of garlic-derived metabolites in milk samples obtained after consumption of roasted garlic. Among the three metabolites investigated in this work, only AMS is responsible for the garlic-like odor of breast milk (12). The AMS concentrations should thus be related to the perceived odor intensities. This was, however, not the case for all milk samples. For example, regarding MR2, the AMS concentration dropped significantly after 2 h, whereas the garlic-like odor of MR2 kept a relatively stable value over the whole sampling time. In case of MR3, the concentration of AMS increased until 4 h after garlic ingestion, then dropped only slightly, but the odor intensity was highest at 4 h and decreased significantly afterwards. The AMS concentrations of MC4 peaked at 2 h but the odor was rated maximal at 4 h. These slight inconsistencies might be related to the fact that the overall garlic odor was of overall low intensity. Moreover, it turned out that the garlic-like odor, especially if faint, faded quickly. In sum, the results demonstrate that the odor of milk is altered after ingestion of roasted and cooked garlic. These changes may be well-perceivable by the infant and might influence its sucking behavior (11) or later food preferences (20). In all three cases, i.e., for raw, roasted, and cooked garlic, AMS was identified as the odor-active principle beyond the garlic-like odor in milk. Despite it is common knowledge that roasted garlic evinces a more nutty and roasted aroma than raw garlic, the sensory-analytical screening of the milk samples revealed no additional odor-active molecules to be present in milk samples after ingestion of roasted or cooked garlic. We therefore conclude that AMS is responsible for garlic-induced alteration of milk odor, irrespective of whether raw, roasted, or cooked garlic is ingested by the mother.

In the present study, we assessed the influence of common culinary processing steps on garlic-related aroma transfer into milk. Future studies should extend our insights to fried garlic and to more realistic consumption scenarios. Garlic is normally part of a dish, and the protein or fat content of the dish might influence metabolic and absorptive processes (6). Therefore, future studies should investigate uptake, metabolization and excretion profiles of garlic constituents in a real-life consumption scenario, e.g., using the example of a Mediterranean diet. These studies should also include a bigger cohort to allow for statistically relevant conclusions about inter-individual differences in the temporal and quantitative profiles of metabolite excretion, and for identifying influencing factors such as gender, age, frequency of garlic intake, or body mass index. From an analytical point of view, complementary headspace techniques or an untargeted GCxGC-MS approach might be employed to screen for potential further garlic-derived metabolites. Finally, the presence of the garlic-like smelling AMS and the odorless metabolites AMSO and AMSO_2_ in milk raise questions with regard to their potential bioactivity in the infant. Therefore, future studies should investigate whether AMS, AMSO, and AMSO_2_ are of physiological relevance to the infant.

## Data Availability Statement

The datasets presented in this article are not readily available because of privacy and data protection. Requests to access the datasets should be directed to Helene M. Loos (helene.loos@fau.de).

## Ethics Statement

The studies involving human participants were reviewed and approved by the Ethical Committee of Friedrich-Alexander-Universität Erlangen-Nürnberg. The participants provided their written informed consent to participate in this study.

## Author Contributions

LS, CS, and AB conceived and designed the experiments. WQ, KH, MP, and PB performed the experiments and analyzed the data. HL, LS, and WQ conceived the publication. All authors have read and approved the final manuscript and are accountable for the content of the work.

## Conflict of Interest

The authors declare that the research was conducted in the absence of any commercial or financial relationships that could be construed as a potential conflict of interest.

## References

[1] Rivlin RS. Historical perspective on the use of garlic. J Nutri. (2001) 131:951S–4. 10.1093/jn/131.3.951S 11238795

[2] Block E The organosulfur chemistry of the genus allium – implications for the organic chemistry of sulfur. Angew Chem Int Edn Eng. (1992) 31:1135–78. 10.1002/anie.199211351

[3] Borek C. Antioxidant health effects of aged garlic extract. J Nutri. (2001) 131:1010s–5. 10.1093/jn/131.3.1010S 11238807

[4] Salehi B Zucca P Orhan IE Azzini E Adetunji CO Mohammed SA Allicin and health: a comprehensive review. Trends Food Sci Technol. (2019) 86:502–16. 10.1016/j.tifs.2019.03.003

[5] Shang A Cao SY Xu XY Gan RY Tang GY Corke H. Bioactive compounds and biological functions of garlic (*Allium sativum* L.). Foods. (2019) 8:246. 10.3390/foods8070246 31284512 PMC6678835

[6] Lawson LD Hunsaker SM. Allicin bioavailability and bioequivalence from garlic supplements and garlic foods. Nutrients. (2018) 10:812. 10.3390/nu10070812 29937536 PMC6073756

[7] Minami T Boku T Inada K Morita M Okazaki Y Odor components of human breath after the ingestion of grated raw garlic. J Food Sci. (1989) 54:763–763. 10.1111/j.1365-2621.1989.tb04703.x

[8] Taucher J Hansel A Jordan A Lindinger W Analysis of compounds in human breath after ingestion of garlic using proton-transfer-reaction mass spectrometry. J Agri Food Chem. (1996) 44:3778–82. 10.1021/jf960640e

[9] Suarez F Springfield J Furne J Levitt M. Differentiation of mouth versus gut as site of origin of odoriferous breath gases after garlic ingestion. Am J Physiol Gastroint Liver Physiol. (1999) 276:G425–30. 10.1152/ajpgi.1999.276.2.G425 9950816

[10] Scheffler L Sauermann Y Heinlein A Sharapa C Buettner A Detection of volatile metabolites derived from garlic (*Allium sativum*) in human urine. Metabolites. (2016) 6:43 10.3390/metabo6040043 PMC5192449 27916960

[11] Mennella JA Beauchamp GK. Maternal diet alters the sensory qualities of human milk and the nursling's behavior. Pediatrics. (1991) 88:737–44. 1896276

[12] Scheffler L Sauermann Y Zeh G Hauf K Heinlein A Sharapa C Detection of volatile metabolites of garlic in human breast milk. Metabolites. (2016) 6:18 10.3390/metabo6020018 PMC4931549 27275838

[13] Mennella JA Johnson A Beauchamp GK. Garlic ingestion by pregnant women alters the odor of amniotic fluid. Chem Senses. (1995) 20:207–9. 10.1093/chemse/20.2.207 7583013

[14] Blankenhorn MA Richards CE Garlic breath odor. J Am Med Assoc. (1936) 107:409–10. 10.1001/jama.1936.02770320013004

[15] Hansanugrum A Barringer SA. Effect of milk on the deodorization of malodorous breath after garlic ingestion. J Food Sci. (2010) 75:C549–58. 10.1111/j.1750-3841.2010.01715.x 20722910

[16] Scheffler L Sharapa C Buettner A. Quantification of volatile metabolites derived from garlic in human breast milk. Food Chem. (2019) 274:603–10. 10.1016/j.foodchem.2018.09.039 30372984

[17] Schaal B Doucet S Soussignan R Rietdorf M Weibchen G Francke W The human breast as a scent organ: Exocrine structures, secretions, volatile components, and possible functions in breastfeeding interactions. In: Hurst JL Beynon RJ Roberts SC Wyatt TD, editors. Chemical Signals in Vertebrates. New York, NY: Springer (2008). p. 325–35.

[18] Mennella JA Beauchamp GK. The transfer of alcohol to human milk - effects on flavor and the infant's behavior. N Engl J Med. (1991) 325:981–5. 10.1056/Nejm199110033251401 1886634

[19] Mennella JA Beauchamp GK The human infants' response to vanilla flavors in mother's milk and formula. Infant Behav Dev. (1996) 19:13–9.

[20] Spahn JM Callahan EH Spill MK Wong YP Benjamin-Neelon SE Birch L. Influence of maternal diet on flavor transfer to amniotic fluid and breast milk and children's responses: a systematic review. Am J Clin Nutri. (2019) 109:1003S–26. 10.1093/ajcn/nqy240 30982867

[21] Mennella JA Beauchamp GK. The effects of repeated exposure to garlic-flavored milk on the nursling's behavior. Pediatr Res. (1993) 34:805–8. 8108198 10.1203/00006450-199312000-00022

[22] Lawson LD Wang ZJ. Allicin and allicin-derived garlic compounds increase breath acetone through allyl methyl sulfide: use in measuring allicin bioavailability. J Agri Food Chem. (2005) 53:1974–83. 10.1021/jf048323s 15769123

[23] Scheffler L Sharapa C Buettner A Quantification of volatile metabolites derived from garlic (*Allium sativum*) in human urine. Front Nutri. (2019) 6:43 10.3389/fnut.2019.00043 PMC6499206 31111029

[24] Bartzatt R Blum D Nagel D Isolation of garlic derived sulfur compounds from urine. Analy Lett. (1992) 25:1217–24. 10.1080/00032719208016123

[25] Cavagnaro PF Camargo A Galmarini CR Simon PW. Effect of cooking on garlic (*Allium sativum* L.) antiplatelet activity and thiosulfinates content. J Agri Food Chem. (2007) 55:1280–8. 10.1021/jf062587s 17256959

[26] Locatelli DA Altamirano JC Gonzalez RE Camargo AB Home-cooked garlic remains a healthy food. J Funct Foods. (2015) 16:1–8. 10.1016/j.jff.2015.04.012

[27] Engel W Bahr W Schieberle P Solvent assisted flavour evaporation - a new and versatile technique for the careful and direct isolation of aroma compounds from complex food matrices. Eur Food Res Technol. (1999) 209:237–41.

[28] van den Dool H Kratz P. A generalization of the retention index system including linear temperature programmed gas-liquid partition chromatography. J Chromatograp A. (1963) 11:463–71. 10.1016/S0021-9673(01)80947-X 14062605

[29] Schieberle P Grosch W Quantitative analysis of aroma compounds in wheat and rye bread crusts using a stable isotope-dilution assay. J Agri Food Chem. (1987) 35:252–7. 10.1021/jf00074a021

[30] Scheffler L Sharapa C Amar T Buettner A. Identification and quantification of volatile ramson-derived metabolites in humans. Front Chem. (2018) 6:410. 10.3389/fchem.2018.00410 30255016 PMC6141758

[31] Egen-Schwind C Eckard R Kemper FH. Metabolism of garlic constituents in the isolated perfused rat liver^*^. Planta Med. (1992) 58:301–5. 10.1055/s-2006-961471 1438588

[32] Lawson LD Wang ZJ Pre-hepatic fate of the organosulfur compounds derived from garlic (*Allium sativum*). Planta Med. (1993) 59:A688–9. 10.1055/s-2006-959976

[33] Germain E Auger J Ginies C Siess MH Teyssier C. *In vivo* metabolism of diallyl disulphide in the rat: identification of two new metabolites. Xenobiotica. (2002) 32:1127–38. 10.1080/0049825021000017902 12593760

[34] Hoebler C Karinthi A Devaux MF Guillon F Gallant DJG Bouchet B. Physical and chemical transformations of cereal food during oral digestion in human subjects. Br J Nutri. (1998) 80:429–36. 10.1017/S0007114598001494 9924264

[35] Canon F Neiers F Guichard E. Saliva and flavor perception: perspectives. J Agri Food Chem. (2018) 66:7873–9. 10.1021/acs.jafc.8b01998 29962207

[36] Dalhamn T Edfors ML Rylander R. Mouth absorption of various compounds in cigarette smoke. Arch Environ Health. (1968) 16:831–5. 10.1080/00039896.1968.10665162 5654556

[37] Harris D Robinson JR. Drug delivery via the mucous membranes of the oral cavity. J Pharmaceut Sci. (1992) 81:1–10. 10.1002/jps.2600810102 1619560

[38] Miron T Rabinkov A Mirelman D Wilchek M Weiner L. The mode of action of allicin: its ready permeability through phospholipid membranes may contribute to its biological activity. Biochim Biophys Acta. (2000) 1463:20–30. 10.1016/S0005-2736(99)00174-1 10631291

[39] Wyss M Kaddurah-Daouk R. Creatine and creatinine metabolism. Physiol Rev. (2000) 80:1107–213. 10.1152/physrev.2000.80.3.1107 10893433

[40] Hare RS. Endogenous creatinine in serum and urine. Proc Soc Exper Biol Med. (1950) 74:148–51. 10.3181/00379727-74-17837 15430417

[41] Owen JA Iggo B Scandrett FJ Stewart CP The determination of creatinine in plasma or serum, and in urine; a critical examination. Biochem J. (1954) 58:426–37. 10.1042/bj0580426 13208633 PMC1269917

[42] Achari R Mayersohn M Conrad KA. HPLC analysis of creatinine in human plasma and urine. J Chromatograp Sci. (1983) 21:278–81. 10.1093/chromsci/21.6.278 6874876

[43] Barr DB Wilder LC Caudill SP Gonzalez AJ Needham LL Pirkle JL. Urinary creatinine concentrations in the U.S. Population: implications for urinary biologic monitoring measurements. Environ Health Perspect. (2005) 113:192–200. 10.1289/ehp.7337 15687057 PMC1277864

[44] Middleton DRS Watts MJ Lark RM Milne CJ Polya DA. Assessing urinary flow rate, creatinine, osmolality and other hydration adjustment methods for urinary biomonitoring using NHANES arsenic, iodine, lead and cadmium data. Environ Health. (2016) 15:68. 10.1186/s12940-016-0152-x 27286873 PMC4902931

[45] Yu TH Wu CM Ho CT Volatile compounds of deep-oil fried, microwave-heated and oven-baked garlic slices. J Agri Food Chem. (1993) 41:800–5. 10.1021/jf00029a023

[46] Yu TH Wu CM Rosen RT Hartman TG Ho CT Volatile compounds generated from thermal degradation of alliin and deoxyalliin in an aqueous solution. J Agri Food Chem. (1994) 42:146–53. 10.1021/jf00037a026

