# Supplementary material for: Quantification of Allyl Methyl Sulfide, Allyl Methyl Sulfoxide, and Allyl Methyl Sulfone in Human Milk and Urine After Ingestion of Cooked and Roasted Garlic
Source: Front Nutr. 2020 Sep 18;7:565496. doi: 10.3389/fnut.2020.565496 (PMC7531236; doi:10.3389/fnut.2020.565496)
Supplement: Supplementary file 2 [file Data_Sheet_1.PDF]

Dietary protocol

Time period:

Date of donation:

		Food	Beverages
DAY 1	Breakfast		
	Z1		
	Lunch		
	Z2		
	Dinner		
	Z3		
DAY 2	Breakfast		
	Z1		
	Lunch		
	Z2		
	Dinner		
	Z3		

DAY OF DONATION	Breakfast		
	Z1		
	Lunch		
	Z2		
	Dinner		
	Z3		

Z1, Z2, Z3 = snack
